# High accuracy distinction of shockable and non-shockable arrhythmias in abnormal classes through wavelet transform with pseudo differential like operators

**DOI:** 10.1038/s41598-023-36463-z

**Published:** 2023-06-12

**Authors:** Md. Masudur Rahman, Sergio Albeverio, Toshinao Kagawa, Shuji Kawasaki, Takayuki Okai, Hidetoshi Oya, Yumi Yahagi, Minoru W. Yoshida

**Affiliations:** 1 Graduate School of Engineering, Kanagawa University, Yokohama, Japan; 2 Inst. Angewandte Mathematik, and HCM, University of Bonn, Bonn, Germany; 3 School of General Education and Management Studies, Suwa University of Science, Nagano, Japan; 4 Faculty of Science and Engineering, Iwate University, Iwate, Japan; 5 Faculty of Information Engineering, Tokyo City University, Tokyo, Japan; 6 Department of Information Systems, Tokyo City University, Tokyo, Japan

**Keywords:** Biomedical engineering, Mathematics and computing

## Abstract

Arrhythmia is an abnormal rhythm of the heart which leads to sudden death. Among these arrhythmias, some are shockable, and some are non-shockable arrhythmias with external defibrillation. The automated external defibrillator (AED) is used as the automated arrhythmia diagnosis system and requires an accurate and rapid decision to increase the survival rate. Therefore, a precise and quick decision by the AED has become essential in improving the survival rate. This paper presents an arrhythmia diagnosis system for the AED by engineering methods and generalized function theories. In the arrhythmia diagnosis system, the proposed wavelet transform with pseudo-differential like operators-based method effectively generates a distinguishable scalogram for the shockable and non-shockable arrhythmia in the abnormal class signals, which leads to the decision algorithm getting the best distinction. Then, a new quality parameter is introduced to get more details by quantizing the statistical features on the scalogram. Finally, design a simple AED shock and non-shock advice method by following this information to improve the precision and rapid decision. Here, an adequate topology (metric function) is adopted to the space of the scatter plot, where we can give different scales to select the best area of the scatter plot for the test sample. As a consequence, the proposed decision method gives the highest accuracy and rapid decision between shockable and non-shockable arrhythmias. The proposed arrhythmia diagnosis system increases the accuracy to 97.98%, with a gain of 11.75% compared to the conventional approach in the abnormal class signals. Therefore, the proposed method contributes an additional 11.75% possibility for increasing the survival rate. The proposed arrhythmia diagnosis system is general and could be applied to distinguish different arrhythmia-based applications. Also, each contribution could be used independently in various applications.

## Introduction

Fatal arrhythmias sometimes cause sudden cardiac death. These arrhythmias are identified by analyzing electrocardiogram (ECG) signals. The ECG provides us with a non-invasive way of diagnosing heart conditions^1^. With a very high rate of sudden death, cardiovascular diseases are observed. Indeed, the statistics reported by World Health Organization indicate clearly that cardiac arrhythmia is the main reason, with 32% of sudden death, over the world^2^. In particular, more than 50,000 people die due to sudden cardiac arrest in Japan^3^, while 50% of the deaths in Europe are caused by cardiovascular diseases^4^.

The automated external defibrillator (AED) is used for arrhythmia patients for first aid. Appropriate use of the AED improves the survival rate^5^. In the first stage of the AED operation, the ECG signals are analyzed to judge whether the AED’s defibrillation should be applied. The important problem here is distinguishing shockable and non-shockable arrhythmia precisely in the abnormal class of the ECG signals. Of the abnormal class, ventricular fibrillation (VF) and ventricular tachycardia (VT) are shockable arrhythmias that require defibrillation to restart the heart for normal electrical function. In contrast, defibrillation must not be applied for pulseless electrical activity (PEA), which is a non-shockable arrhythmia. If AED applied the shock to the patient with PEA arrhythmia, it would harm the patient’s heart^6^. Therefore, AED’s precision and quick decision for the discrimination between the shockable and non-shockable arrhythmia in the abnormal class is crucially important. As for the quickness, note that the survival rate decreases from 7 to 10% per minute according to the statistics of the American heart association (AHA) and resuscitation academy^7,8^.

In order to increase the precision of AED, it is necessary to extract accurate information from abnormal ECG signals. Many researchers^9–13^ analyzed the ECG signals in the time-frequency domain based on wavelet transform. The main advantage of wavelet transform is observing the ECG signals’ behaviors in the time and frequency domain simultaneously through the scalogram. It is well known that the time-frequency resolution in the Short-Time Fourier Transform (STFT), Wigner-Ville Distribution function (WVD), Polynomial WVD (PWVD) methods, and so on, is constant over time and frequency^14–16^. On the other hand, the time-frequency window in Continuous Wavelet Transform (CWT) is considered as a parameter, and thus it automatically adjusted to low-frequency motion for a long period of time and high-frequency motion for a short period of time. Namely, CWT can be obtained the optimal time-frequency resolution by changing the time resolution according to the frequency of the signal component. Thus, we adopt CWT for the analysis of ECGs. The successful works^9,11^ apply just a standard Gabor wavelet transform (GWT) to generate a scalogram from the ECG signal, which gives a good distinction between normal and abnormal signals. However, it does not achieve enough discrimination between shockable and non-shockable arrhythmias in the abnormal classes (PEA, VF, and VT). This issue is addressed by the novel method in Rahman et al.^17^, where the wavelet transforms with pseudo-differential-like operators were applied to observe statistics on the scalogram of the ECG signal. However, the proposed method is implemented for equally spaced time intervals to derive the scalogram since the unequally spaced wavelet function is not defined.

The function *L*(*a*), called pseudo-differential-like operators in Eq. (3), is a modulator of wavelet transform *Wf*(*a*, *b*) that works just like a Fourier multiplier. It controls the *Wf*(*a*, *b*) by multiplying a larger or smaller value when we would like to emphasize or suppress the scale components in question, respectively. Note that we have tried to take all possible factors of the pseudo-differential-like operators of the frequency in the experiments. For example, we have taken the power of the frequency, the inverse of the frequency, the multiplication of the scale factor with the frequency, etc. Then, we perform the qualitative and quantitative evaluation, from which we select the best pair of pseudo-differential-like operators with a non-linear transformation function. As a related method of time-frequency decomposition of signals, the least-squares wavelet analysis (LSWA) is known^18^. In the LSWA, weighted least-squares wavelet spectrogram (LSWS), a normalized weighted quadratic form of wavelet coefficients is considered, and its effectiveness in the signal analysis is demonstrated. Actually, the quadratic form is a cross-correlation between the original signal and its least-square approximation in the sense of a normal equation for time-frequency components of the original signal. Merit of the LSWA is that it can be used for non-equi-spaced time signals, as well as it has a higher time-frequency resolution. The weights make it possible to control the effect of being non-equi-space, smoothing out the values on the irregular time instants to regular instants by the weights. It is applied successfully in VLBI antenna signal analysis where signals are basically supposed to be non-equi-spaced^19^. As a result of the LSWS analysis, the most or least significant antenna sites of annual coherency are clearly identified, in a superior way to classical LS spectral analysis, using a useful software called LSWAVE software (http://www.ghader.org/lswave.html, and https://github.com/Ghaderpour/LSWAVE-SignalProcessing/). The LSWAVE is an open-access signal analysis tool with a graphical user interface (GUI) that includes the least-squares spectral analysis to the least-squares cross-wavelet analysis.

So far, the scalogram analysis has been mainly considered only along the frequency axis. However, we can draw out more information from the scalogram of the ECG signals, which is useful for better discrimination, by characterizing the scalogram in the time-frequency plane. To the best of our knowledge, there have been no attempts to characterize the scalogram in the time-frequency plane^17,20,21^. In the present paper, we independently observe the scalogram’s behaviors along the time and frequency axes. This makes it possible to quantify the different statistical features on the scalogram of the abnormal class signals.

The decision algorithm determines if the patient has a life-threatening arrhythmia and makes a shock or no-shock decision. Therefore, the decision algorithm is a crucial factor in the safety and performance of an AED. The decision method should be designed by considering the characteristics of each problem. Many researchers apply the different types of decision algorithms (e.g., Mahalanobis distance, nearest neighbor, etc.) to distinguish the arrhythmias in the decision stage^11,22–25^. However, blindly use of such general methods is not the best for considering our problems. For example, the decision through the Mahalanobis distance depends on the concept of an approximation using the Gaussian distributions. Although the nearest neighbor is a simple, non-parametric decision method, and evaluation is performed by the Euclidean distance, but this Euclidean metric function-based decision method has an issue with selecting the number of neighbors of the test sample. The decision becomes changed for selecting the different number of neighbors. Also, overfitting and underfitting occur for choosing the number of one nearest neighbor and the total number of data of nearest neighbors of the test sample. We can mitigate this issue by adopting adequate topology (a new metric function) to the space of the scatter plot. In addition, researchers use machine learning classifier^26–36^ in the decision stage where a large number of the dataset is required and a substantial computation time to generate the decision is not practical for diagnosis purposes. Therefore, an accurate and rapid decision method for the AED shock and non-shock advice algorithm is the ultimate demand to use the scalogram information properly. For a viable solution to the above issues, we develop a simple decision method (design of the AED shock and non-shock advice algorithm) that guarantees high distinction with a low computational amount.

The main contributions of this paper are as follows:

(i) **Derivation of the scalogram.** By making use of the Gabor wavelet transform with pseudo-differential-like operators, developed in^17^, the time-frequency scalograms of the ECG corresponding to abnormal shockable (VT and VF) and abnormal non-shockable (PEA) arrhythmias are generated. The main novelty of the proposed method in^17^ is that the application of pseudo-differential like operators with non-linear transformation function to the GWT does work efficiently and effectively, and generates distinguishable scalograms between shockable and non-shockable arrhythmias in the abnormal class signals, which satisfy visual comparison through scalo-graphic and scatter plot observation.
(ii) **Effective characterization of the scalogram in both time and frequency direction.** In this context, we apply two quality parameters, normalized spectrum index (NSI) and normalized time index (NTI), in the scalogram. The NSI possesses the information in the frequency direction, which has been considered in Rahman et al.^17^. On the other hand, the NTI possesses the information in the time direction, which is a new addition to this paper.
(iii) We employed the class separability technique to select essential features effective for discrimination.
(iv) **Design of the AED shock and non-shock advice algorithm.** We develop a simple decision method to guarantee high accuracy and rapid decision between shockable and non-shockable arrhythmias. In this context, we adopt a new metric function, defined through adequately chosen topology for the space of scatter plots. The main novelty of the proposed decision method is that it effectively discriminates between shockable and non-shockable arrhythmias with low computational time, which help to increase the survival rate of the patients, and the application of the proposed metric function in the decision method achieves the highest accuracy than the application of the Euclidean metric function in the decision method.
(v) We conducted a comparative performance analysis of our proposed methodology with other state-of-the-art approaches. It is shown that the proposed arrhythmia diagnosis system performs better than others for the distinction between abnormal shockable (VT, VF) and abnormal non-shockable (PEA) arrhythmias in the abnormal class signals.

## The proposed methodology of the arrhythmia diagnosis system

**Figure 1:**
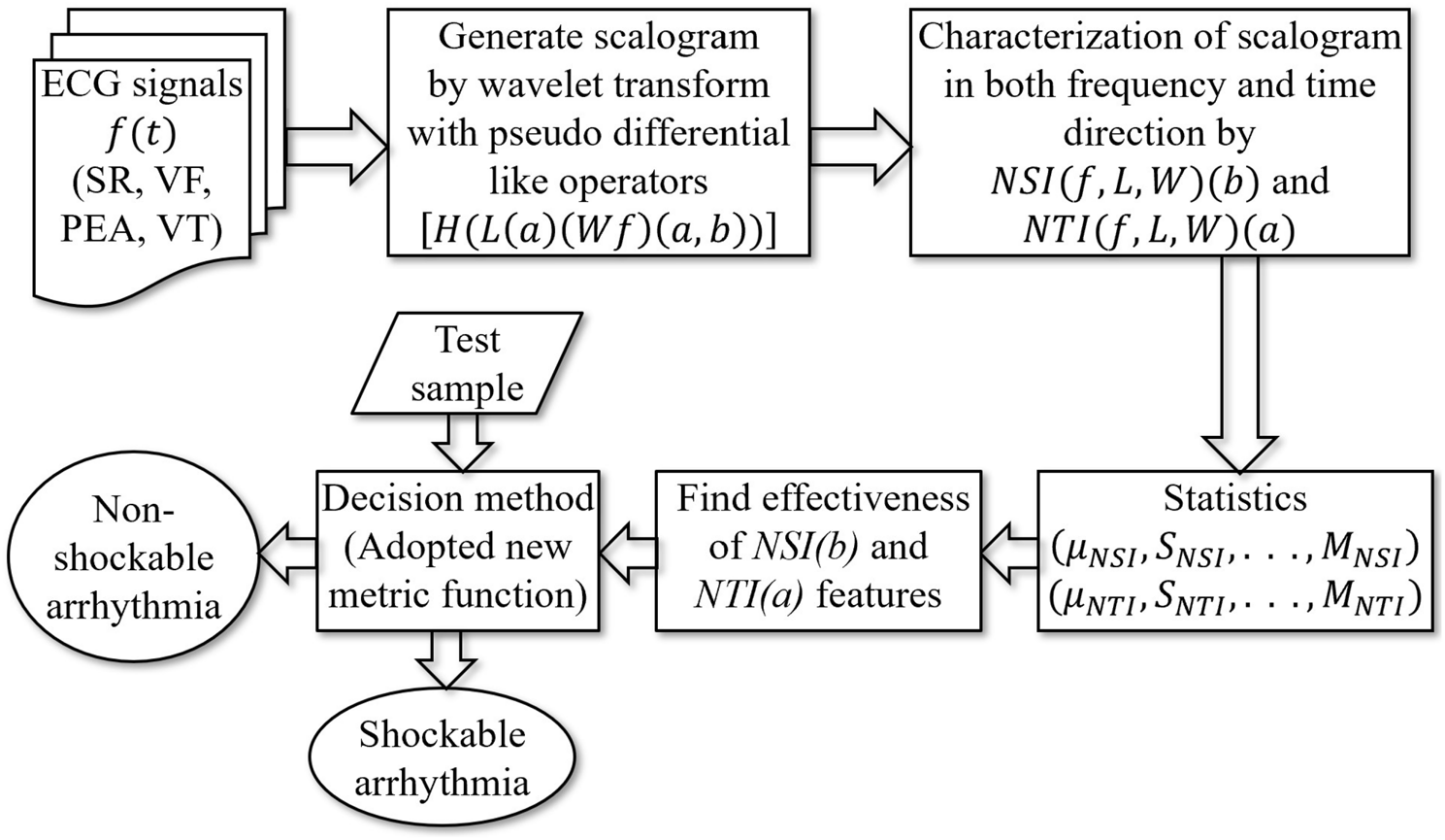
The whole scheme of shockable and non-shockable arrhythmia discrimination.

The proposed discrimination procedure consists of several steps shown in Fig. 1.

This figure describes the overall summary of the distinction process between shockable and non-shockable arrhythmias. In the proposed arrhythmia diagnosis system, the core idea is to derive exact information (scalogram) from the abnormal classes of ECG signals, which leads to the decision algorithm for accurate discrimination between shockable and non-shockable arrhythmias. Following the information, the new quality parameter is adopted to get more details by quantizing the statistical features. Also, a method is proposed in the decision stage to get high accuracy and rapid discrimination, increasing the chance of survival. First, the pre-processing of the ECG signals for segmenting and detrending is performed. The original ECG signals are separated into pieces of five-second signal segments. Then, the trend is removed from each of the segmented signals to obtain the signals *f*. Second, the wavelet transforms with pseudo-differential-like operators, and non-linear transformation is used to accurately generate wavelet coefficients *H*(*L*(*a*)*Wf*(*a*, *b*)) from *f*. These coefficients are represented as a scalogram. Third, as a basic statistic to quantize the different features over the abnormal class ECG signals of the scalogram, we take the NSI^17^ and NTI. We then extracted 16 statistical features from the scalogram through NSI and NTI. Fourth, to determine the effective features, we watch each generated feature independently and test their discriminatory capabilities using the class separability technique. The three features with the highest score are selected and depicted in a three-dimensional scatter plot for group-wise discrimination. The last stage is shockable and non-shockable arrhythmia discrimination which is performed using the proposed method. A test sample is classified based on an open neighbor with the minimum distance by adopting a new metric function in the method.

### ECG dataset

**Figure 2:**
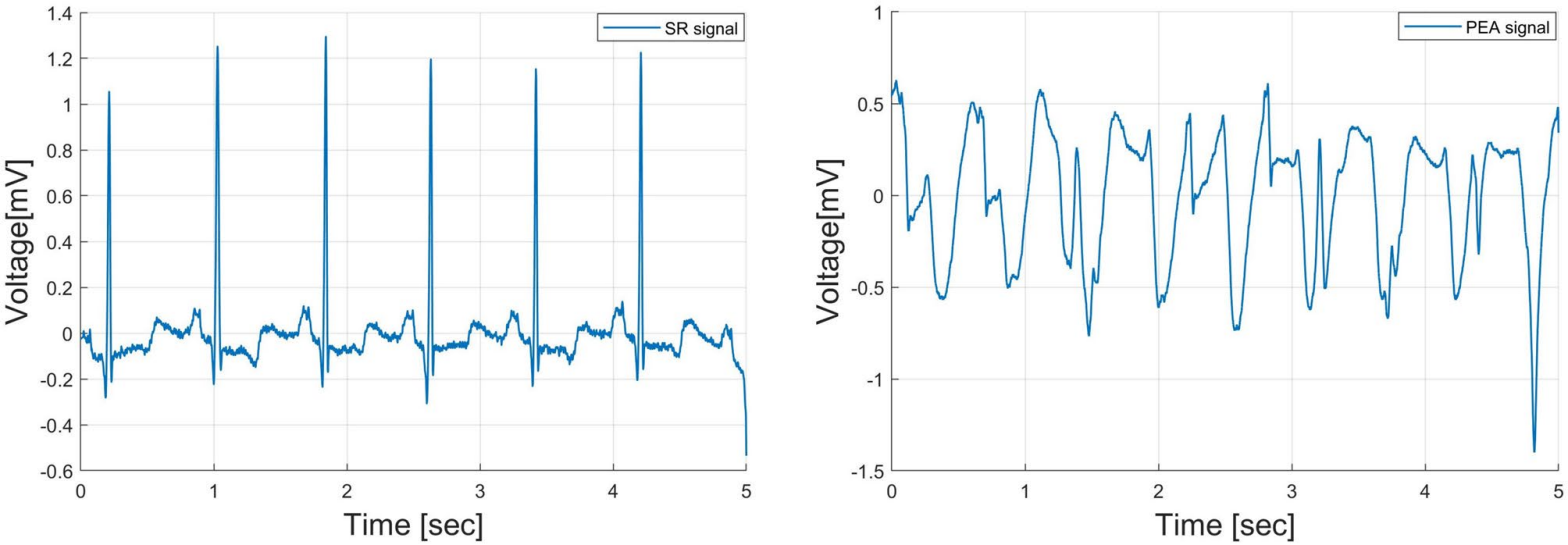
An example of non-shockable ECG (SR: left, PEA: right).

**Figure 3:**
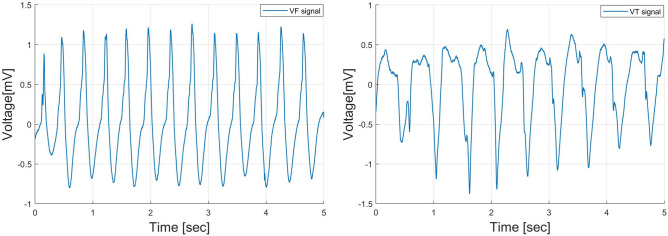
An example of shockable ECG (VF: left, VT: right).

A combination of three accredited databases from physionet.org^37^, MIT-BIH arrhythmia database (MITDB), MIT-BIH malignant ventricular ectopy database (VFDB), and Creighton university ventricular tachyarrhythmia database (CUDB), is used to examine the proposed method for distinction of the shockable and non-shockable arrhythmia signals. These databases contain both shockable and non-shockable arrhythmia types. From these databases, 1079 ECG samples are collected. The samples are grouped into four classes: sinus rhythm (SR), pulseless electrical activity (PEA), ventricular fibrillation (VF), and ventricular tachycardia (VT). The pre-processing of the data set has been explained in^17^. Figures 2 and 3 show an example of the segmented non-shockable and shockable arrhythmias, respectively. In Fig. 2, the left is SR, and the right is PEA, respectively, both of non-shockable arrhythmias. On the other hand, in Fig. 3 the left is VF, and the right is VT, respectively, both shockable arrhythmias.

### The wavelet transform with pseudo-differential like operators

In order to give a self-consistent style to the present paper and to avoid the confusion of the notions corresponding to the pseudo differential operators, we repeat the description given in^17^. The notions of the pseudo-differential like operators have been proposed by Rahman et al., in^17^ to apply it to the analysis of the ECG signals, and the general notions of the pseudo differential operators in the framework of the Fourier analysis are well-known and are found in^38^. Moreover, general applications of the pseudo differential operators to function spaces can be found, e.g., in^39^ and^40^ and references therein. The usual pseudo-differential operator is defined in the framework of the Fourier analysis. We extend the notion of the pseudo-differential operator to the wavelet analysis framework and call them the pseudo-differential-like operators. The notion of the pseudo differential operators to the GWT is defined as follows:   Let $L^2 \equiv L^2({{\mathbb {R}} } \rightarrow {{\mathbb {C}}})$ be the space of ${{\mathbb {C}}}$-valued, complex number valued, square-integrable functions on the real line ${{\mathbb {R}}}$. For some given $\sigma >0$ and $\omega _0 \in {{\mathbb {R}}}$, take the mother wavelet function $\psi (t)$ in $L^2$ as follows:

1 $$\begin{aligned} \psi (t) \equiv \frac{1}{\sqrt{2 \pi \sigma ^2}} e^{- \frac{t^2}{2 \sigma ^2}} e^{i \omega _0 t}, \quad t \in {{\mathbb {R}}}, \text{ with } \, i \equiv \sqrt{-1}. \end{aligned}$$

Then, for $f \in L^2$, define the Gabor wavelet transform (*Wf*)(*a*, *b*) as follows:

2 $$\begin{aligned} (Wf)(a,b) \equiv \frac{1}{\sqrt{a}} \int _{- \infty }^{\infty } f(t) {\overline{\psi \left( \frac{t-b}{a}\right) }} dt, \quad a > 0, \quad b \in {{\mathbb {R}}}, \end{aligned}$$

where, the variable $\frac{1}{a} >0$ corresponds to the frequency of the function *f*, and *b* corresponds to the time. Next, we prepare two measurable functions *L* and *H* such that

$$\begin{aligned} L\, : \, {{\mathbb {R}}}_{+} \ni a \longmapsto L(a) \in {{\mathbb {C}}}, \quad H \, : \, {{\mathbb {C}}} \ni y \longmapsto H(y) \in {{\mathbb {C}}}. \end{aligned}$$

For $f \in L^2$, we then define our wavelet transform with pseudo differential like operator *L*, and its non-linear transform by means of *H*, which are ${{\mathbb {C}}}$-valued measurable functions with the variables $a > 0$ and $b \in {{\mathbb {R}}}$, as follows:

3 $$\begin{aligned} L(a) \cdot (Wf)(a,b), \quad H\bigg ( L(a) \cdot (Wf)(a,b) \bigg ). \end{aligned}$$

**Figure 4:**
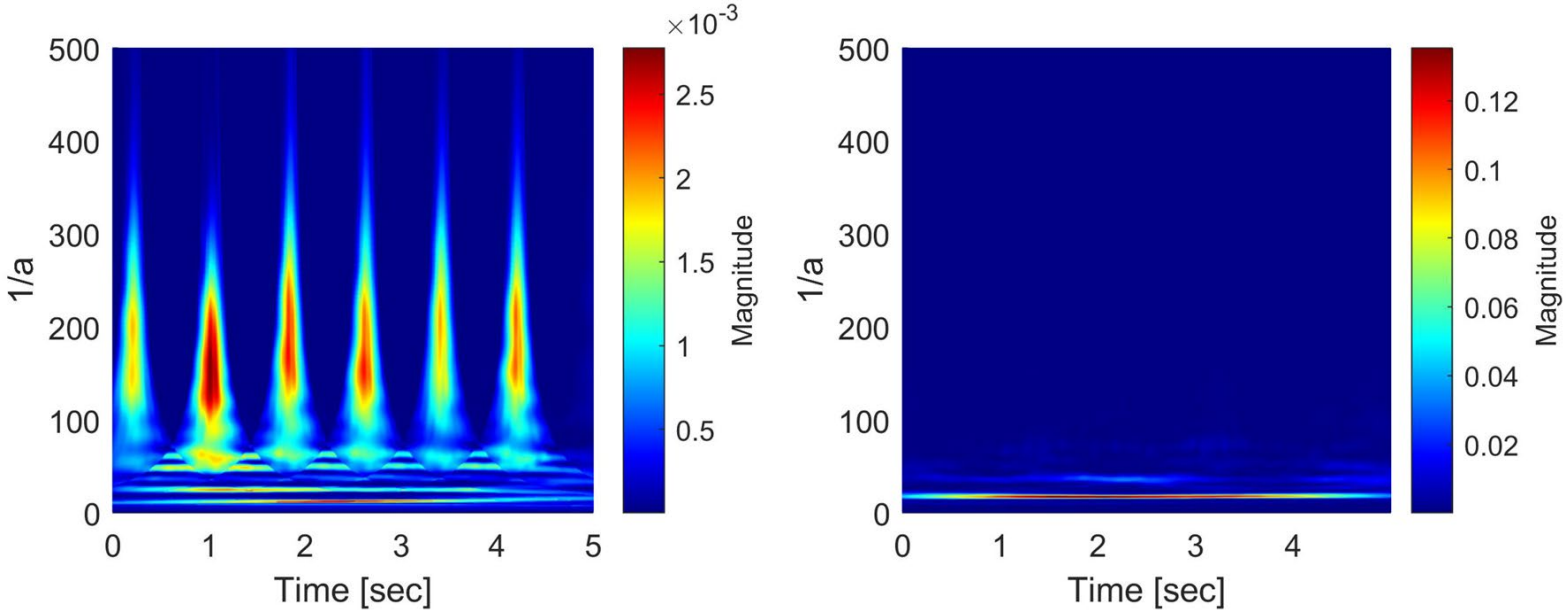
Generated scalograms by setting $L(a) = 1$ with $H(\cdot )={|{\cdot }|}^2$ (SR: left, PEA: right).

**Figure 5:**
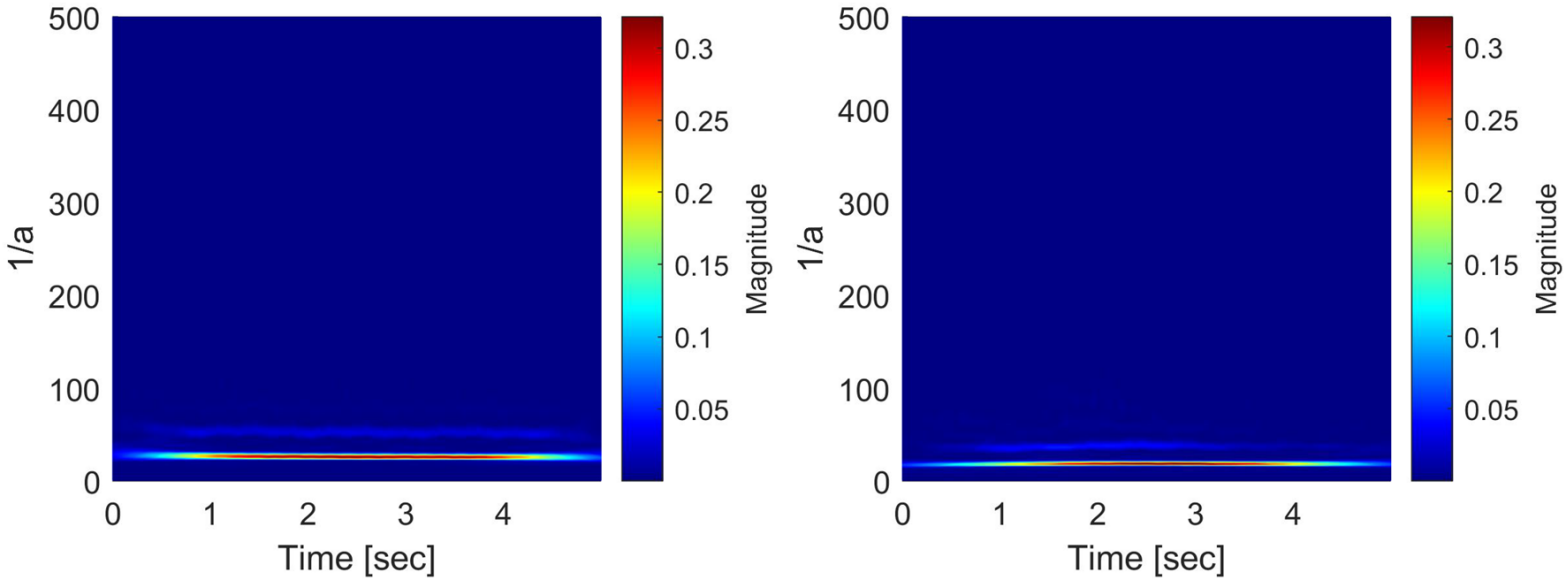
Generated scalograms by setting $L(a) = 1$ with $H(\cdot )={|{\cdot }|}^2$ (VF: left, VT: right).

**Figure 6:**
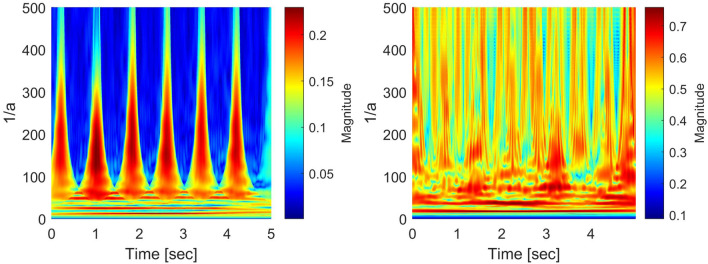
Generated scalograms by setting $L(a)=\frac{1}{a}$ with $H(\cdot )={|{\cdot }|}^{\frac{1}{4}}$ (SR: left, PEA: right).

**Figure 7:**
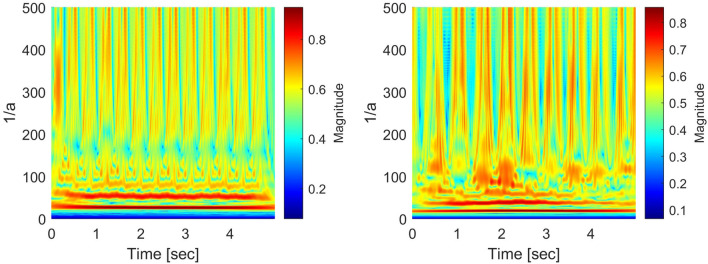
Generated scalograms by setting $L(a)=\frac{1}{a}$ with $H(\cdot )={|{\cdot }|}^{\frac{1}{4}}$ (VF: left, VT: right).

The algorithm 1 shows the process of generating the scalogram using the Gabor wavelet transform with pseudo-differential-like operators. From Figs. 4, 5, 6 and 7, through the source code by which these figures are derived, the vertical axis values can be interpreted as [Hz] by multiplying $1/\pi$.
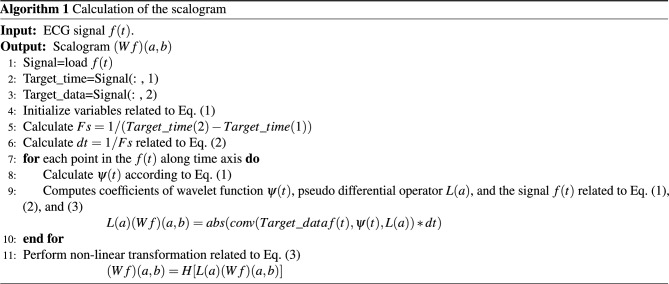

Let us find how the application of the pseudo-differential like operators is powerful to the delicate distinctions of shockable and non-shockable arrhythmia in abnormal classes.

(i) Figures 4, 5 (which are given in^17^), In these figures the scalograms with $L(a) = 1$ and $H(\cdot ) = {|{\cdot }|}^2$, which is the conventional setting adopted by^9^, where the pseudo differential like operators are not applied. They show a good distinction between normal and abnormal signals. Still, there seem to be no differences in the scalo-graphic representation between abnormal signals, i.e., PEA, VF, and VT. In the scalograms, wavelet coefficient values for all scalograms are at the same level, which leads to failure to get the best distinction in the decision algorithm.
(ii) Figures 6, 7 (which are given in^17^), In these figures, the scalograms with $L(a) = \frac{1}{a}$ and $H(\cdot )= {|{\cdot }|}^{\frac{1}{4}}$, which is the setting of pseudo-differential like operators and non-linear transformation. Through the pseudo-differential like operators, we can get much more fruitful information (fractional order of differentiation of the signal) on the original signals, and by applying the non-linear transformation functions to the transformed signals, we can make bigger the part of the transformed signals which has small amplitude. Through these, we can clearly distinguish the signals with small differences, i.e., PEA, VF, and VT. In particular, the difference between the maximum frequencies corresponding to PEA and VT is 7.2 (Hz) (randomly selected samples). The different values over time lead to getting the best discrimination in the decision stage.

We have demonstrated an intrinsic effect of *L*(*a*) with $H(\cdot )$ using qualitative evaluation in^17^. From the experimental results (cf.^17^), for the subsequent considerations, we adopt the pseudo-differential like operators $L(a) = \frac{1}{a}$ with the non-linear transformation $H(\cdot ) = {|{\cdot }|}^{\frac{1}{4}}$. In^17^, the scalograms corresponding to $L(a) = a$ and $H(\cdot ) = {|{\cdot }|}^{\frac{1}{4}}$;   $L(a) = (\frac{1}{a})^2$ and $H(\cdot ) = {|{\cdot }|}^{\frac{1}{4}}$;   $L(a) = (\frac{1}{a})^{\frac{1}{2}}$ and $H(\cdot ) = {|{\cdot }|}^{\frac{1}{4}}$   are also derived and considered in detail.

### Effective characterization of scalogram

Here, we present how to use the scalogram in the time and frequency plane to calculate statistical features effective for discrimination.

#### Characterization along with the frequency

**Figure 8:**
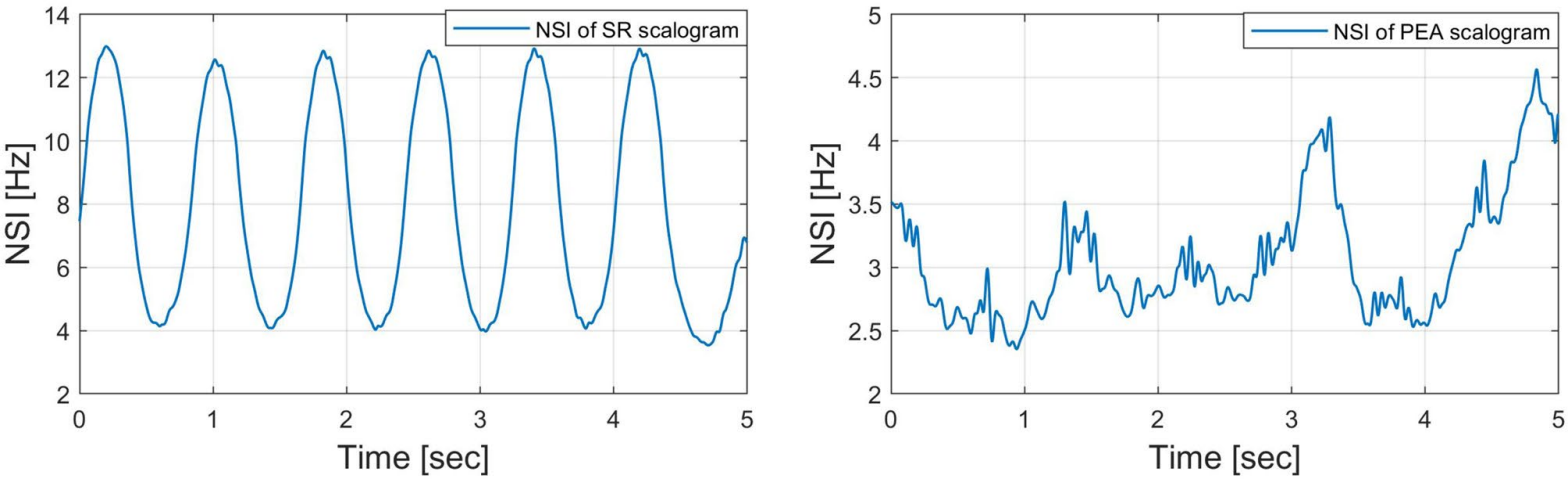
*NSI*(*b*) of scalograms (SR: left, PEA: right).

**Figure 9:**
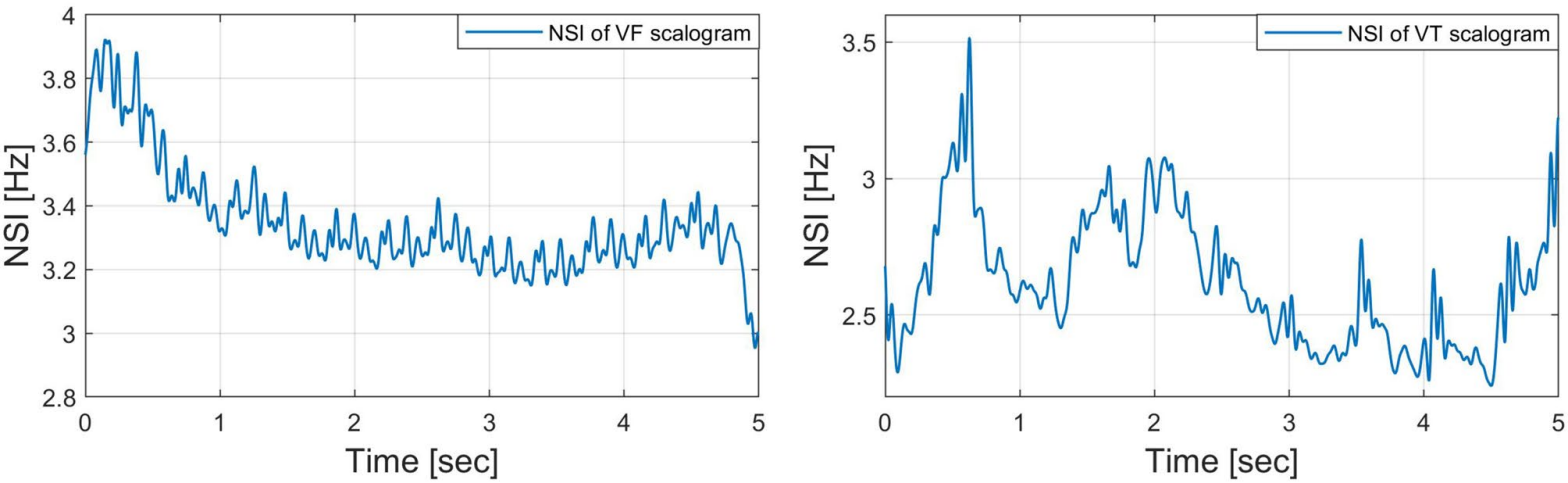
*NSI*(*b*) of scalograms (VF: left, VT: right).

For the scalogram analysis along with the frequency, we have adopted the quality parameter NSI in^17^ and taken the center of gravity of energies over frequencies of the scalogram. Figures 8 and 9 show an example of NSI that was obtained as a “time series” signal from the scalogram for SR, PEA, VF, and VT signals. The visualization shows that the NSI waveform tends to change periodically and regularly for an SR signal, while the changes are irregular for PEA, VF, and VT signals. In the example, we mainly concentrate on the discrimination of the shockable (VF and VT) and non-shockable (PEA) arrhythmia in the abnormal class through the NSI. Hence, the NSI value over time is the primary key here. Inspecting the maximum, we get different NSI values for PEA VF and VT signals. The definition of NSI is as follows:

4 $$\begin{aligned} NSI(b)\equiv \frac{\sum _{a}{E(a,b)}F(a)}{\sum _{a}{E(a,b)}}, \end{aligned}$$

where *E*(*a*, *b*) and *F*(*a*) represent scalogram value and scalogram frequency, respectively. Note that *E*(*a*, *b*) in the scalogram is obtained by *H*(*L*(*a*) (*Wf*)(*a*, *b*)). The frequency *F*(*a*) is for the corresponding *E*(*a*, *b*). Algorithm 2 shows the characterization method of the scalogram over the frequency.
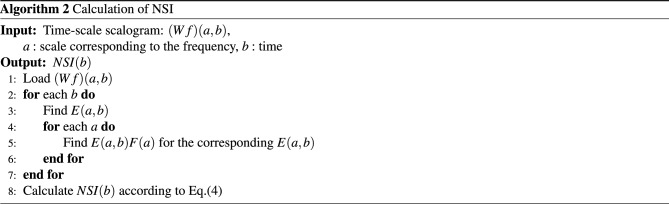

#### Characterization along with the time

**Figure 10:**
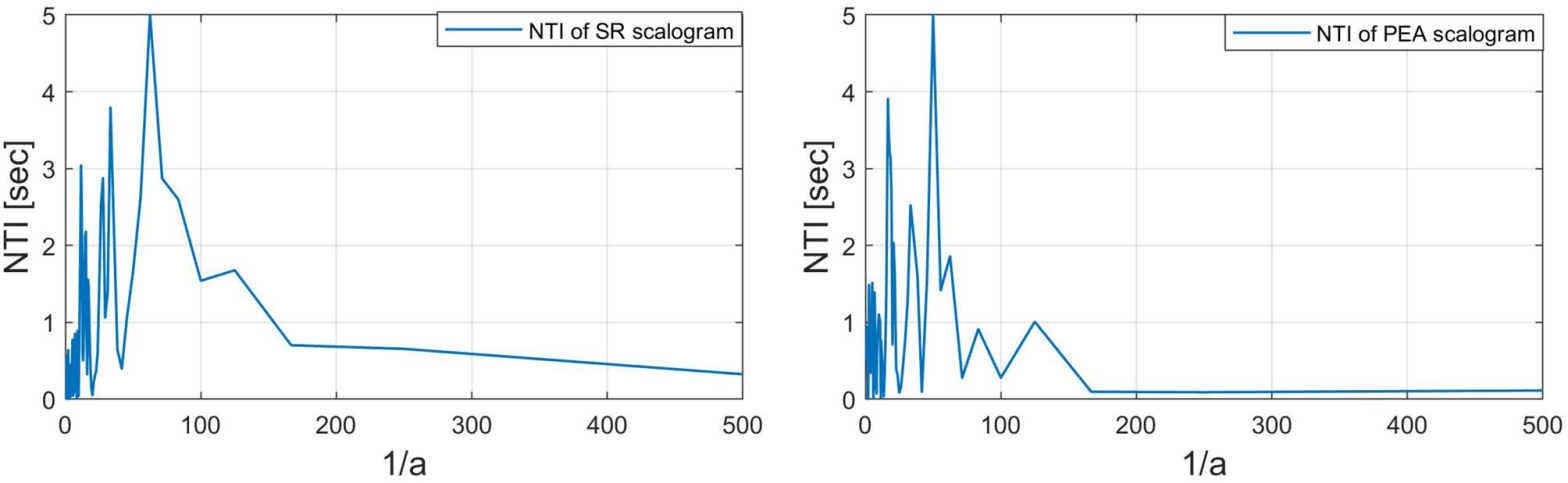
*NTI*(*a*) of scalograms (SR: left, PEA: right).

**Figure 11:**
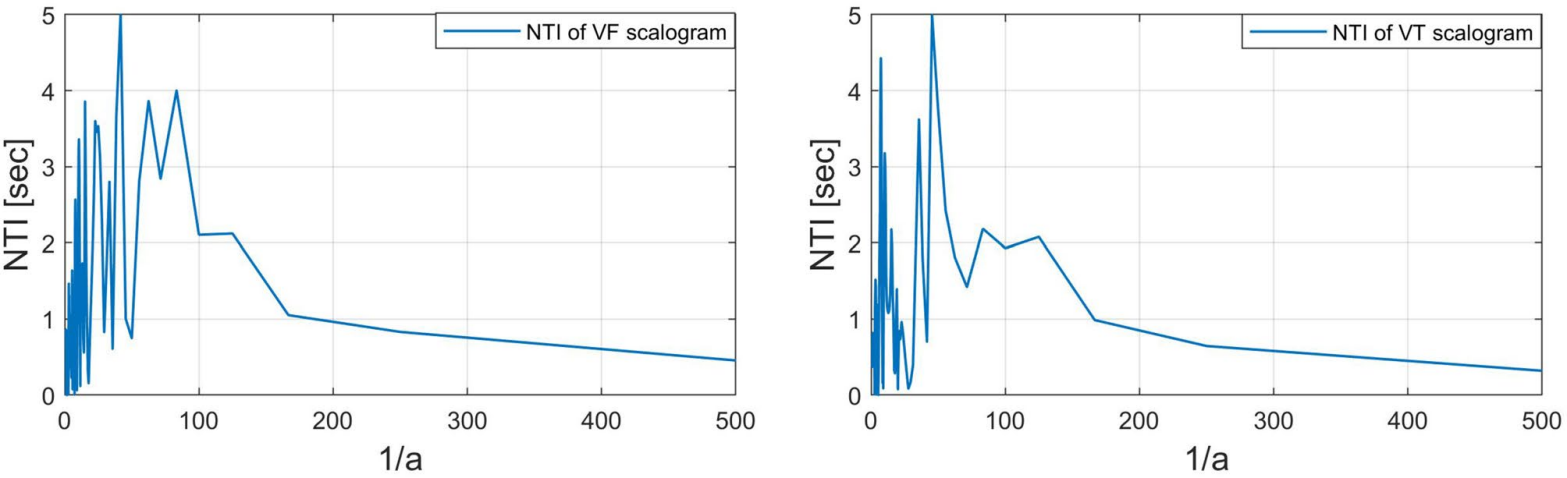
*NTI*(*a*) of scalograms (VF: left, VT: right).

We also adopt a new quality parameter NTI for additional analysis of the scalogram along the time direction. The NTI gives the center of gravity of energies over time of the scalogram. The NTI for SR, PEA, VF, and VT signals are shown in Figs. 10 and 11. Here, the NTI is obtained as a waveform over frequencies from the scalogram. We observe that the NTI spectrum is different for all classes of arrhythmia. The different NTI spectrum for each class lead to good discrimination in the decision algorithm. The definition of the NTI is given by

5 $$\begin{aligned} NTI(a)\equiv \frac{\sum _{b}{E(a,b)}T(b)}{\sum _{b}{E(a,b)}}, \end{aligned}$$

where *E*(*a*, *b*) and *T*(*b*) represent scalogram value and scalogram time, respectively. Note that *E*(*a*, *b*) in the scalogram obtained by *H*(*L*(*a*) (*Wf*)(*a*, *b*)) and the time *T*(*b*) is for the corresponding *E*(*a*, *b*). The algorithm 3 shows the procedure to characterize the scalogram along the time direction.
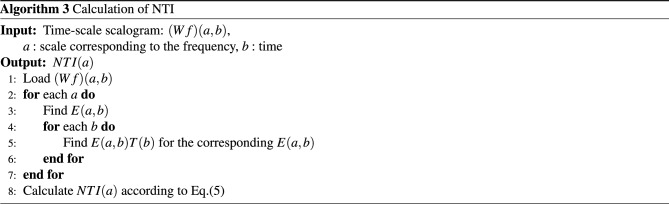

### Effectiveness of the *NSI*(*b*) and *NTI*(*a*) features

Based on the NSI and NTI of the scalogram, a total of sixteen statistical features are derived. Tables 1 and 2 show the features extracted from the scalogram through NSI and NTI. It is unclear here which features are effective for discriminating shockable and non-shockable arrhythmias. Therefore, it is necessary to find out the discriminatory abilities of features. In order to find out the effective features, we watch each of the generated features independently and test their discriminatory capabilities by using the class separability technique such as scatter matrices^41^. This procedure helps us to select the best feature from the set of features. Algorithm 4 shows a detailed process to find the effective feature.

**Figure 12:**
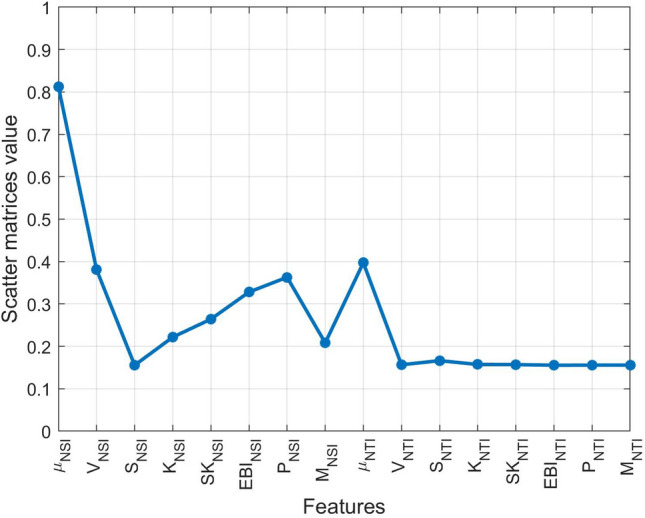
Discriminatory capabilities of individual features for multi-class separation.

**Figure 13:**
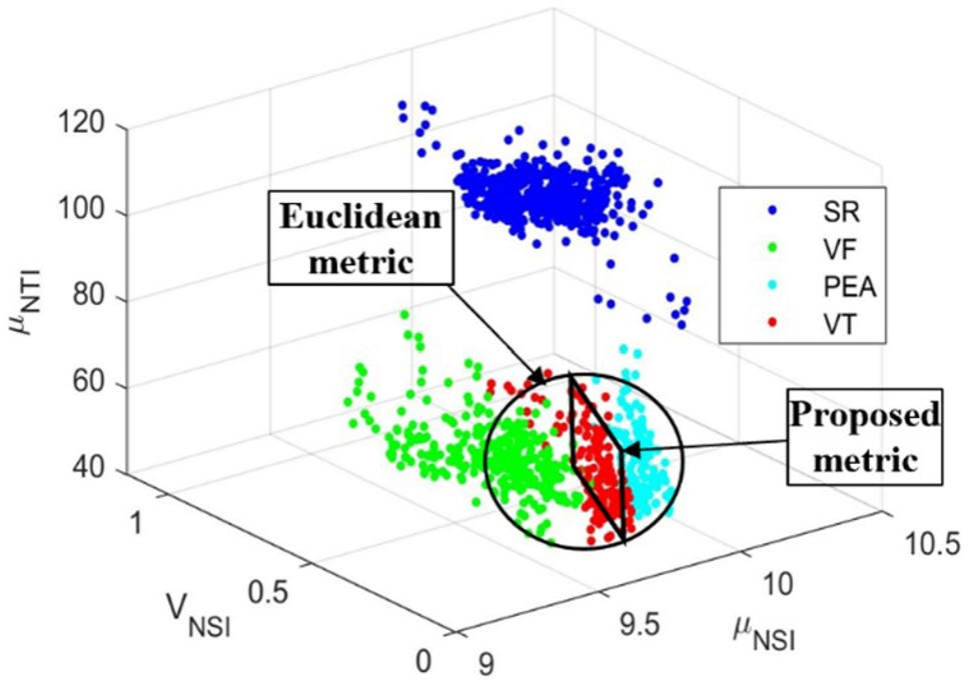
3D scatter plot of the best three features.

Suppose that we have an n-dimensional feature vector $\bar{x}=[x_{1},x_{2},\dots ,x_{n}]$ assigned to *c* different classes $(i=1,2,\dots ,c)$. The definition of within-class scatter matrix $S_w$ and between-class scatter matrix $S_b$ are given by, respectively:

6 $$\begin{aligned} S_{w}=\sum _{i=1}^{c} \sum _{x\epsilon D_{i}} P_{i}(x-\mu _{i})(x-\mu _{i})^{T}, \end{aligned}$$

7 $$\begin{aligned} S_{b}=\sum _{i=1}^{c}P_{i}(\mu _{i}-\mu )(\mu _{i}-\mu )^{T}, \end{aligned}$$

where $D_i$ is the *i*th class, and $P_i$ is a priori probability for class $D_i$. That is $P_{i}={n_{i}}/{N}$, where $n_i$ is the number of samples in class $D_i$, out of a total of *N* samples. The classwise mean $\mu _i$ and the overall mean $\mu$ are defined by:

8 $$\begin{aligned} \mu _{i}=\frac{1}{n_{i}}\sum _{x\epsilon D_{i}}x, \end{aligned}$$

9 $$\begin{aligned} \mu =\frac{1}{N} \sum _{D}x, \end{aligned}$$

respectively, where *D* is the set of all classes. The scatter matrices value in Fig. 12 provides insight into how the separation between the four different arrhythmias is using the individual features. In the figure, the feature “mean of NSI“has the highest scatter matrices value, indicating that this feature has the best discriminatory capabilities. Also, the “mean of NTI“ and “variance of NSI“ have the second-best discriminatory capability, whereas the rest of the features are less than a satisfactory level. The selected best three features are visualized by 3D scatter plot that displays the good separation corresponding to the abnormal groups and the group-wise distribution is very much scattered, where the proposed metric function fitted well on the scatter plot than the Euclidean metric function (see Fig. 13).

**Table 1:** List of features derived through *NSI*(*b*).

No.	Feature name	Symbol
1	Mean of NSI	$\mu _{NSI}$
2	Variance of NSI	${\mathcal {V}}_{NSI}$
3	Slope of NSI	${\mathcal {S}}_{NSI}$
4	Kurtosis of NSI	${\mathcal {K}}_{NSI}$
5	Skewness of NSI	${{\mathcal{S}\mathcal{K}}}_{NSI}$
6	Entropy of NSI	${{\mathcal {EBI}}}_{NSI}$
7	Power of NSI	${\mathcal {P}}_{NSI}$
8	Mode of NSI	$M_{NSI}$

**Table 2:** List of features derived through *NTI*(*a*).

No.	Feature name	Symbol
1	Mean of NTI	$\mu _{NTI}$
2	Variance of NTI	${\mathcal {V}}_{NTI}$
3	Slope of NTI	${\mathcal {S}}_{NTI}$
4	Kurtosis of NTI	${\mathcal {K}}_{NTI}$
5	Skewness of NTI	${{\mathcal{S}\mathcal{K}}}_{NTI}$
6	Entropy of NTI	${{\mathcal {EBI}}}_{NTI}$
7	Power of NTI	${\mathcal {P}}_{NTI}$
8	Mode of NTI	$M_{NTI}$

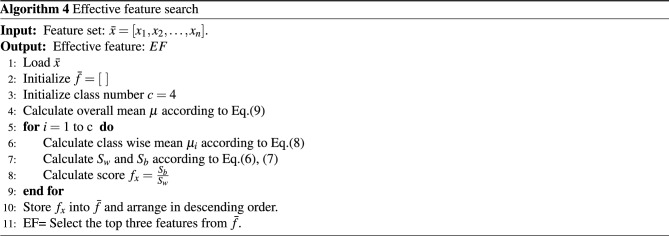



### Topology of the scatter plot on *D* dimensional Euclidean space

We explain the concept of the topology of the scatter plot (see Fig. 13), through which we are able to get a high accuracy distinction between different groups of the arrhythmias. We give the corresponding mathematical description in a concise way and do not go further into the mathematics^42^ (General topology) for the corresponding mathematics. In addition, algorithm  5 shows the detailed process for the discrimination of shockable and non-shockable arrhythmia. Recall that our objective of the present research is to give a high-accuracy distinction procedure by using the information available from the scatter plot. For this purpose, we should choose an adequate topology for the given scatter plot. In the theory of statistics and corresponding mathematical software, there exist several provided methods of classification, e.g., the Mahalanobis distance and nearest neighbor evaluation. However, such provided methods would not always be optimal for each problem in consideration. For example, in the case when we are given a scatter plot on *D* dimensional Euclidean space, then the Mahalanobis distance is defined through the covariance matrix of the scatter plot of training data of a given group, e.g., the group of the ECG signals of SR, etc., which is a real symmetric non-negative definite $D \times D$ matrix by which we can define a multi-variable Gaussian distribution. Hence, the classification through the Mahalanobis distance depends on the concept of an approximation using the Gaussian distributions. Also, the nearest neighbor evaluation is performed by the Euclidean distance, where we can choose more adequately for each problem in consideration.

Suppose that we are given a non-negative function $\rho ({\textbf{x}}, {\textbf{y}})$ on the product space of *D* dimensional Euclidean space ${{\mathbb {R}}}^D \times {{\mathbb {R}}}^D$, ${{\mathbb {R}}} \equiv (- \infty , \infty )$ the real line, that satisfies the following:

$$\begin{aligned} \rho ({\textbf{x}}, {\textbf{y}}) = \rho ({\textbf{y}}, {\textbf{x}})\ge 0, \quad \text{ for } \text{ any } \, {\textbf{x}} \in {{\mathbb {R}}}^D, {\textbf{ y}} \in {{\mathbb {R}}}^{D}, \end{aligned}$$

$$\begin{aligned} \rho ({\textbf{x}}, {\textbf{y}}) =0 \qquad \text{ if } \text{ and } \text{ only } \text{ if } \, \, {\textbf{x}} = {\textbf{y}}. \end{aligned}$$

We note that here we do not ask $\rho$ to be a function that satisfies the triangle inequality such that $\rho ({\textbf{x}}, {\textbf{y}}) \le \rho ({\textbf{x}}, {\textbf{z}}) + \rho ({\textbf{z}}, {\textbf{ y}})$ for any ${\textbf{x}}, \, {\textbf{y}}, \, {\textbf{z}} \, \in {{\mathbb {R}}}^D$, and the $\rho$ does not a metric function in general. For each ${\textbf{x}} \in {{\mathbb { R}}}^D$ and $r >0$, let us define an open neighborhood of the point ${\textbf{x}} \in {{\mathbb {R}}}^D$ as follows:

10 $$\begin{aligned} \mathcal{O}({\textbf{x}}; r) \equiv \{ {\textbf{y}} \in {{\mathbb {R}}}^D \, : \, \rho ({\textbf{x}}, {\textbf{y}}) < r \, \}. \end{aligned}$$

Then, we can define a new topology on ${{\mathbb {R}}}^D \times {{\mathbb {R}}}^D$, which is generated by the open base such that

11 $$\begin{aligned} \{ \mathcal{O}({\textbf{x}}; r) \, : \, {\textbf{ x}} \in {{\mathbb {R}}}^D, \, r >0 \}, \end{aligned}$$

i.e., the family of the open neighbourhood $\mathcal{O}({\textbf{x}}; r)$ defined by equation (10).

**Figure 14:**
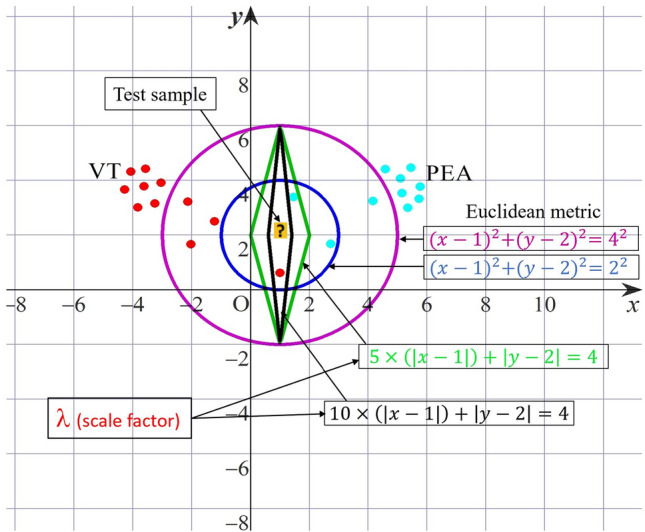
Decision strategy based on open neighborhood topology (Scatter point of training data and neighborhood of test data in two-dimensional case.).

Our distinction procedure adopted here is as follows: Suppose that we are given a scatter plot of training data (see Fig. 13), and test data (we do not know to which group of arrhythmias it belongs), denoted by ${\textbf{x}}$. Take the largest $r >0$ by which $\mathcal{O}({\textbf{x}}; r)$ include only one training data, say ${\textbf{y}}$, namely ${\textbf{ y}}$ is the nearest point to the test data ${\textbf{x}}$ evaluated by $\rho$. Then we decide that the test data ${\textbf{x}}$ is the same group as the one of ${\textbf{y}}$ (see Fig. 14). For some special cases where the nearest points of ${\textbf{x}}$ evaluated by $\rho$ are not only one point, we may prepare an adequate algorithm by which we can avoid ambiguity. As an example, we can take $\rho$ as follows:

12 $$\begin{aligned} \rho ({\textbf{x}}, {\textbf{y}}) \equiv {\lambda }_1 |{x_1 - y_1}|^{p_1} + \cdots + {\lambda }_D |{x_D - y_D}|^{p_D}, \quad \text{ for } \, {\textbf{ x}} = (x_1, \dots , x_D), \, {\textbf{y}} = (y_1, \dots , y_D) \, \in {{\mathbb {R}}}^D, \end{aligned}$$

where ${\lambda }_j,$ $j=1, \dots , D$ and $p_j$, $j=1, \dots , D$ are given positive numbers. More generally, we can take $\rho$ as follows:

13 $$\begin{aligned} \rho ({\textbf{x}}, {\textbf{y}}) \equiv w({\textbf{ x}}, {\textbf{y}}) \, \, A\, \, {}^t w({\textbf{x}}, {\textbf{y}}), \quad with \quad ({\textbf{x}}, {\textbf{y}}) \equiv \left( |{x_1 - y_1}|^{\frac{p_1}{2}}, \dots , |{x_D -y_D}|^{\frac{p_D}{2}} \right) , \end{aligned}$$

where ${}^t w({\textbf{x}}, {\textbf{y}})$ is the transpose of the vector $w({\textbf{x}}, {\textbf{y}})$, and *A* is a real symmetric positive-definite $D\times D$ matrix:

$$\begin{aligned} A = \left( \begin{array}{ccc} a_{11} &{}\quad \ldots &{}\quad a_{1D} \\ \ldots &{}\quad \ldots &{}\quad \ldots \\ a_{D1} &{}\quad \ldots &{}\quad a_{DD} \end{array} \right) , \end{aligned}$$

with real $a_{ij} = a_{ji}$, $i, j = 1, \dots , D$. In particular, by taking *A* as the diagonal matrix of which diagonal elements satisfy $a_{ii} = {\lambda }_i$, $i = 1, \dots , D$, then equation (13) is reduced to (12). Note that for the $\rho$ satisfying the equation (13), the topology defined through (10), and (11) is equivalent to the one defined through the Euclidean metric $d({\textbf{x}}, {\textbf{y}}) = \sqrt{(x_1- y_1)^2 + \cdots + (x_D - y_D)^2 }$, but we evaluate the distance between ${\textbf{x}}$ and ${\textbf{y}}$ by $\rho ({\textbf{x}}, {\textbf{y}})$, not by $d({\textbf{ x}}, {\textbf{y}})$.

In short, by several $\rho$ we can give the different scales to the space of the scatter plots. We should choose a $\rho$ that is adequate to the present distinction problem. In the present paper we put $D=3$ and through the experiment, we choose ${\lambda }_j$, $j=1, 2, 3$ and $p_j$, $j=1, 2, 3$ as follows:

$$\begin{aligned} \lambda _1 = 6, \quad \lambda _2 = 1, \quad \lambda _3 = 1,\quad and \quad p_1 = 1, \quad p_2 = 1, \quad p_3 = 1. \end{aligned}$$

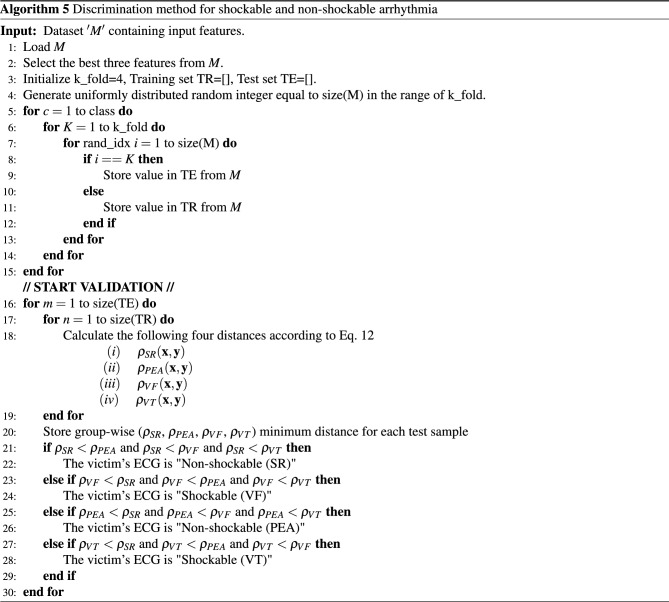


## Performance evaluation and discussion

Here, we explain the evaluation strategy, step by step performance result of our proposed method and compare it with shockable and non-shockable state-of-the-art methods.

### Evaluation matrices

We use macro-and micro-average precision, recall, F1-score (F-measure), and accuracy as performance indices which are commonly used in multi-class classification measurement^43,44^. The F-measure is the harmonic mean of precision and recall. In order to obtain the macro-average F1 score, we compute F-measure $(F_{i})$ for each class and then take their average of F-measure over all classes as:

$$\begin{aligned} F_{i} =2\frac{P_{i}*R_{i}}{P_{i}+R_{i}},\quad Macro-avg.F1=\frac{1}{c}\sum _{i=1}^{c}F_{i}, \end{aligned}$$

where *c* is total number of classes and the precision $(P_{i})$ and recall $(R_{i})$ for class *i* are defined as follows:

$$\begin{aligned} P_{i}=\frac{TP_{i}}{TP_{i}+FP_{i}},\quad R_{i}=\frac{TP_{i}}{TP_{i}+FN_{i}}. \end{aligned}$$

Here $TP_{i}$, $FP_{i}$, and $FN_{i}$ are true positive, false positive, and false negative in the *ith* class, respectively.

The macro average precision $(P_{macro})$ and the macro average recall $(R_{macro})$ are the averages of individual precision and recall respectively:

$$\begin{aligned} P_{macro}=\frac{1}{c}\sum _{i=1}^{c} \frac{TP_{i}}{TP_{i}+FP_{i}}, \quad R_{macro}=\frac{1}{c}\sum _{i=1}^{c} \frac{TP_{i}}{TP_{i}+FN_{i}}. \end{aligned}$$

On the other hand, the micro-average F1 score is given as follows:

$$\begin{aligned} Micro-avg. F1 =2\frac{P_{micro}*R_{micro}}{P_{micro}+R_{micro}}, \end{aligned}$$

where micro average precision $(P_{micro})$ and micro average recall $(R_{micro})$ are computed by summing individual precision and recall as follows

$$\begin{aligned} P_{micro}= \frac{\sum _{i=1}^{c}{TP_{i}}}{\sum _{i=1}^{c}({TP_{i}+FP_{i}})}, \quad R_{micro}= \frac{\sum _{i=1}^{c}{TP_{i}}}{\sum _{i=1}^{c}({TP_{i}+FN_{i}})}. \end{aligned}$$

The accuracy is the ratio of correctly predicted observation to the total observation, that is:

$$\begin{aligned} Accuracy=\frac{TP+TN}{TP+FP+FN+TN}. \end{aligned}$$

### Evaluation process

**Figure 15:**
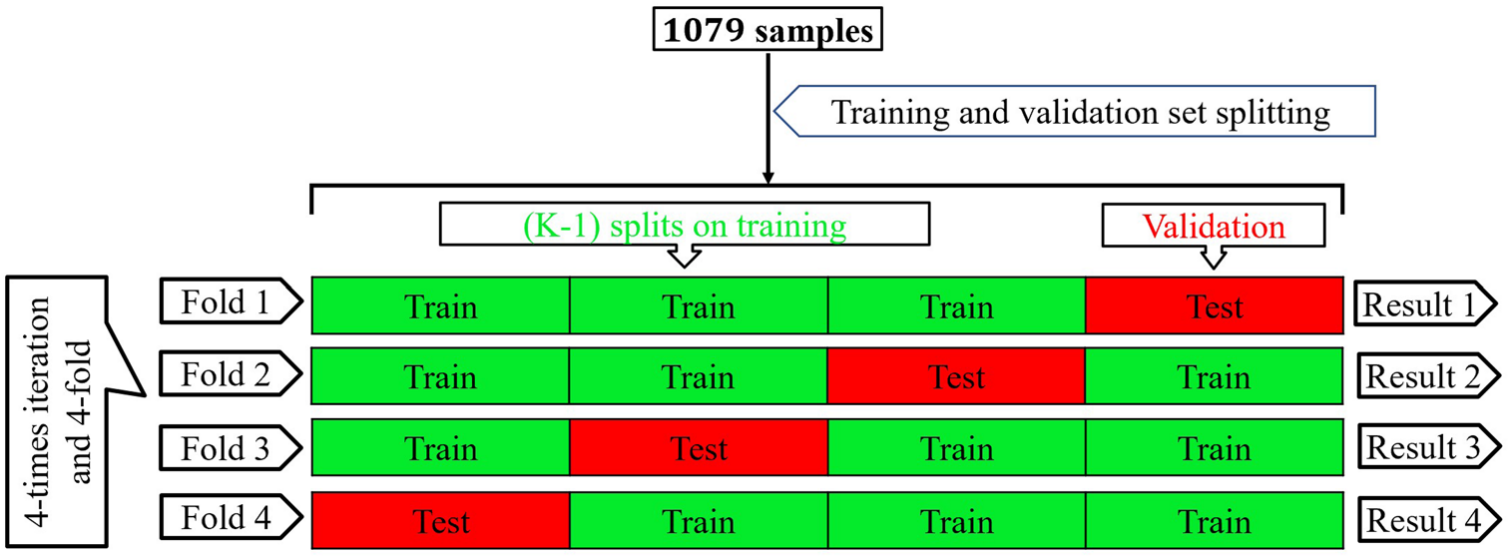
Schematic illustration of four-fold cross-validation approach.

We performed k-fold cross-validation^45^ to stabilize the performance of the proposed method. The discrimination results of each iteration for the 1079 samples are in Tables 3, 4, 5 and 6). We have ${\mathcal {Z}}_{total}=1079$ samples where (SR (Non-shockable) ${\mathcal {Z}}_{total}^{SR}=491)$, (PEA (Non-shockable) ${\mathcal {Z}}_{total}^{PEA}=134)$, (VF (Shockable) ${\mathcal {Z}}_{total}^{VF}=299)$ and (VT (Shockable) ${\mathcal {Z}}_{total}^{VT}=155)$. Here, we have selected k = 4, so the total of $({\mathcal {Z}}_{total}=1079)$ samples are randomly partitioned into four sub-samples of equal size. A single sub-sample, denoted by ${\mathcal {T}}$, is used as the validation data for testing the model, and the remaining $({\mathcal {Z}}_{total}\ -\ {\mathcal {T}})$ sub-samples are used as training data. Here, the ${\mathcal {T}}$ samples are also selected randomly for each type of ECG signal. The cross-validation process is repeated four times, and the process is shown in Fig. 15.

### Performance results

The performance results of the proposed method are evaluated for four class categories using a four-fold cross-validation approach (see “Evaluation process“) based on the evaluation matrices (see “Evaluation matrices“).

**Figure 16:**
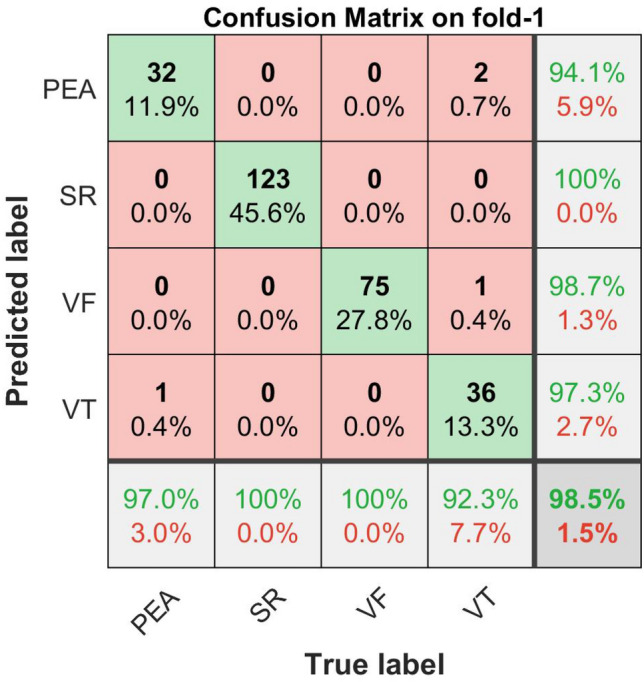
Confusion matrix with performance for shockable and non-shockable arrhythmias on fold-1,($\mu _{NSI}$, ${V}_{NSI}$ and $\mu _{NTI}$, and scale factor, $\lambda _1 = 6,\,\, \lambda _2 = 1, \,\, \lambda _3 = 1$ cases).

**Figure 17:**
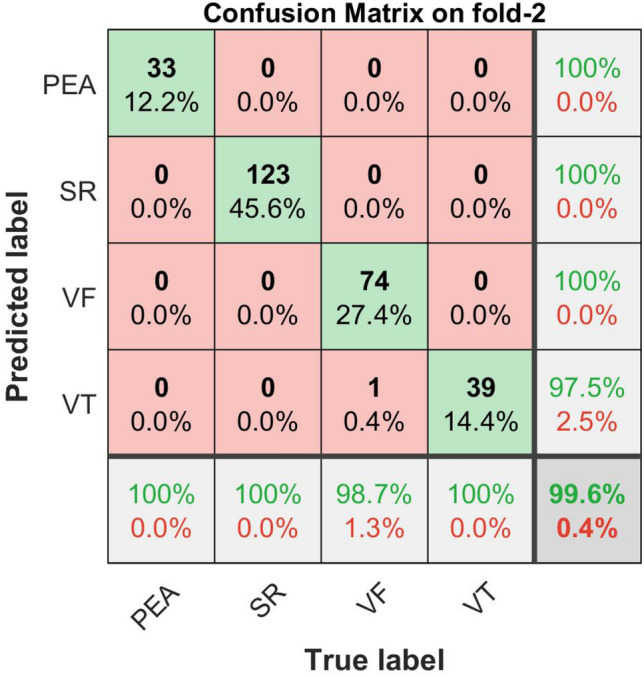
Confusion matrix with performance for shockable and non-shockable arrhythmias on fold-2, ($\mu _{NSI}$, ${V}_{NSI}$ and $\mu _{NTI}$, and scale factor, $\lambda _1 = 6,\,\, \lambda _2 = 1, \,\, \lambda _3 = 1$ cases).

**Figure 18:**
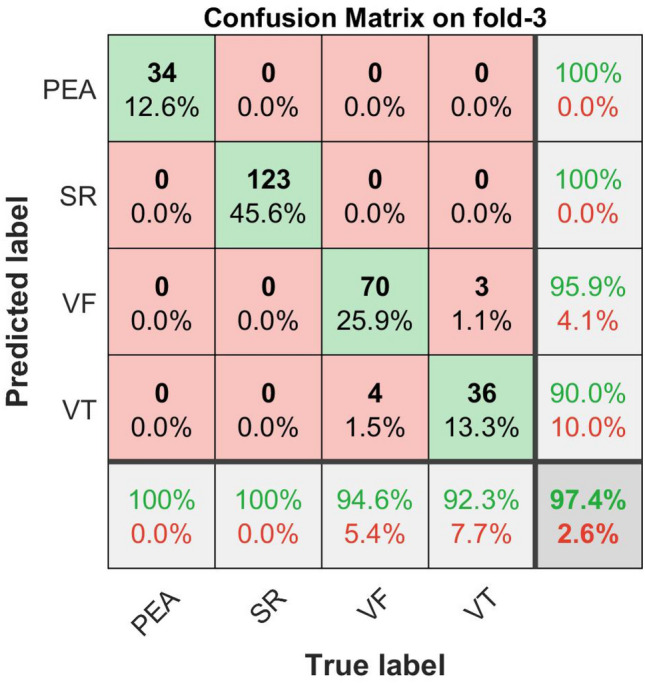
Confusion matrix with performance for shockable and non-shockable arrhythmias on fold-3, ($\mu _{NSI}$, ${V}_{NSI}$ and $\mu _{NTI}$, and scale factor, $\lambda _1 = 6,\,\, \lambda _2 = 1, \,\, \lambda _3 = 1$ cases).

**Figure 19:**
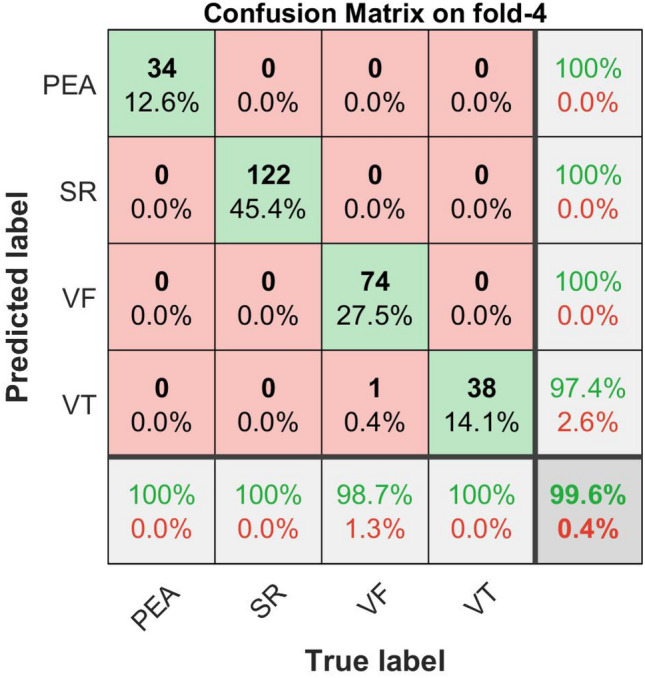
Confusion matrix with performance for shockable and non-shockable arrhythmias on fold-4, ($\mu _{NSI}$, ${V}_{NSI}$ and $\mu _{NTI}$, and scale factor, $\lambda _1 = 6,\,\, \lambda _2 = 1, \,\, \lambda _3 = 1$ cases).

The confusion matrix plots with the performance results for shockable (VF, VT) and non-shockable (SR, PEA) arrhythmias are shown in Figs. 16, 17, 18, and 19 respectively. The confusion matrix is generated through the proposed metric function-based decision method with the scale factor, $\lambda _1 = 6,\,\, \lambda _2 = 1, \,\, \lambda _3 = 1$, by using the combination of the “Mean of NSI“, “Variance of NSI“, and “Mean of NTI“ features. In Figs. 16, 17, 18, and 19, the rows correspond to the predicted class, and the columns correspond to the actual class. The diagonal cells correspond to observations that are correctly classified. The off-diagonal cells correspond to incorrectly classified observations. Both the number of observations and the percentage of the total number of observations are shown in each cell. The values on the far right column (green and red color) and the row at the bottom (green and red color) of each figure show the percentages of correct and incorrect predictions, respectively. The cell in the bottom right of the plot shows the overall correct and incorrect accuracy.

For example, in Fig. 16, 270 data, composed of 33 of PEA, 123 of SR, 75 of VF, and 39 of VT, is tested. The first column shows that the 32 PEA data within the actual 33 PEA test data are correctly identified, and 1 PEA test data is miss judged as VT. Similarly, the second column shows that the actual 123 SR test data are correctly identified, and none of them are misjudged as others, i.e., PEA, VF, or VT. Similarly, the fourth column explains that, within the actual 39 number of VT, 36 are correctly identified, but 1 data is misjudged as VF, and 2 are misjudged as PEA. Therefore, 7.7% incorrect result given at the bottom of the fourth column indicated as the red color is calculated from $(1+2)/(1+2+36) = 3/39$.

On the other hand, the row concern, the first row of the same figure, shows that 32 PEA are exactly identified as PEA, but in addition, 2 of VT are miss judged as PEA, and the far-right component 94.1% of this row, indicated as the green color, is calculated from $32/(32 + 2)$. Similarly, the fourth row shows that 36 VT data are identified correctly, but in addition, 1 of the PEA data is miss judged as VT. Therefore, 97.3% corrected (green color) and 2.7% (red color) incorrect results are calculated from $36/(36+1)$ and $1/(36+1)$, which are shown in the far right of the fourth row. The cell in the bottom right of the plot of the same figure shows the overall 98.5% correct and 1.5% incorrect accuracy.

**Figure 20:**
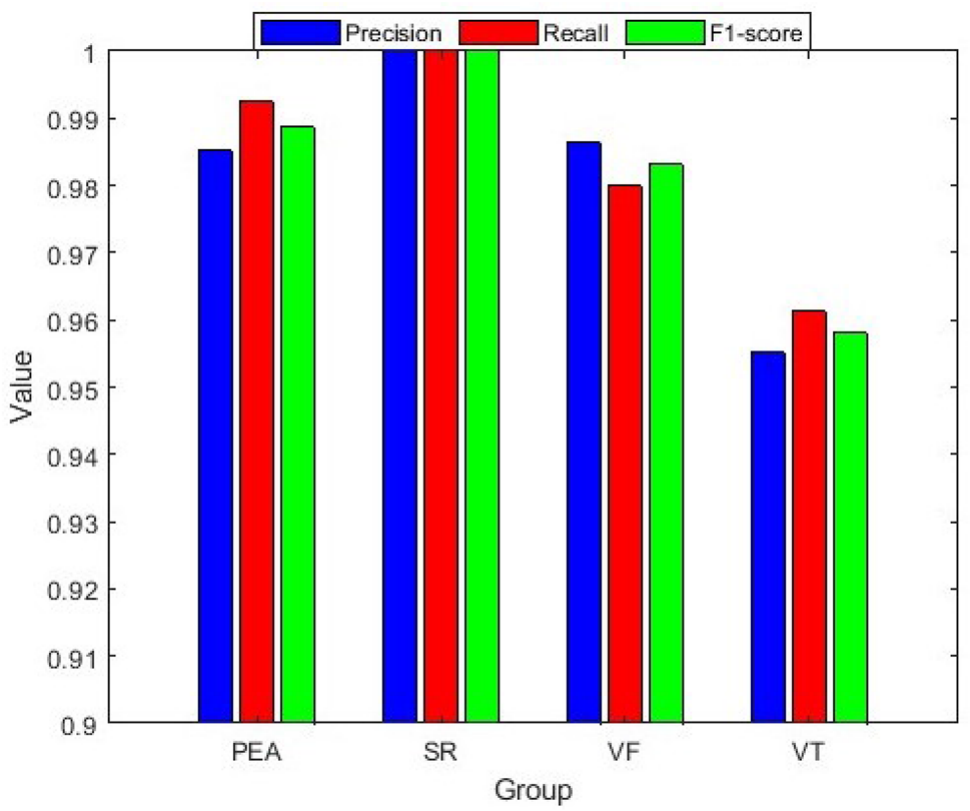
Group-wise precision, recall and F1-score for all test samples.

The detailed performance analysis (fold-wise and group-wise) presented in the Tables 3, 4, 5 and 6, which corresponding to Figs. 16, 17, 18, and 19. The table shows individual precision, recall, F1-score, and accuracy for each group, and shows overall macro and micro average precision, recall, and F1-score. For example, Table 3 presents 0.9412 precision, 0.9697 recall, 0.9552 F1-score, and 98.88% accuracy for PEA test data. Similarly, for SR test data 1.0 precision, 1.0 recall, 1.0 F1-score, and 100% accuracy are obtained, respectively. On the other hand, 0.9868 precision, 1.0 recall, 0.9934 F1-score, and 99.62% accuracy for VF test data and 0.9730 precision, 0.9231 recall, 0.9474 F1-score, and 98.51% accuracy for VT test data are obtained, respectively on fold-1. The overall macro and micro average precision, recall, F1-score of 0.9752, 0.9732, 0.9740, and 0.9852 on fold-1, 0.9938, 0.9967, 0.9952, and 0.9963 on fold-2, 0.9647, 0.9673, 0.9659, and 0.9741 on fold-3, 0.9936, 0.9967, 0.9951, and 0.9963 on fold-4, respectively are shown in Tables 3, 4, 5 and 6. The group-wise precision, recall, and F1-score for all the test samples are illustrated in Fig. 20 where the blue, red, and green bar represents precision, recall, and F1-score, respectively.

**Table 3:** Performance of the proposed method on fold-1, ($\mu _{NSI}$, ${V}_{NSI}$, $\mu _{NTI}$, and scale factor, $\lambda _1 = 6,\,\, \lambda _2 = 1, \,\, \lambda _3 = 1$ cases).

Fold no.	Group	Precision	Recall	F1-score	Accuracy (%)
Fold-1	PEA	0.9412	0.9697	0.9552	98.88
	SR	1.0	1.0	1.0	100.0
	VF	0.9868	1.0	0.9934	99.62
	VT	0.9730	0.9231	0.9474	98.51
	Macro avg.	0.9752	0.9732	0.9740	
	Micro avg.	0.9852	0.9852	0.9852

**Table 4:** Performance of the proposed method on fold-2, ($\mu _{NSI}$, ${V}_{NSI}$, $\mu _{NTI}$, and scale factor, $\lambda _1 = 6,\,\, \lambda _2 = 1, \,\, \lambda _3 = 1$ cases).

Fold no.	Group	Precision	Recall	F1-score	Accuracy (%)
Fold-2	PEA	1.0	1.0	1.0	100.0
	SR	1.0	1.0	1.0	100.0
	VF	1.0	0.9867	0.9933	99.62
	VT	0.9750	1.0	0.9873	99.62
	Macro avg.	0.9938	0.9967	0.9952	
	Micro avg.	0.9963	0.9963	0.9963

**Table 5:** Performance of the proposed method on fold-3, ($\mu _{NSI}$, ${V}_{NSI}$, $\mu _{NTI}$, and scale factor, $\lambda _1 = 6,\,\, \lambda _2 = 1, \,\,\lambda _3 = 1$ cases).

Fold no.	Group	Precision	Recall	F1-score	Accuracy (%)
Fold-3	PEA	1.0	1.0	1.0	100.0
	SR	1.0	1.0	1.0	100.0
	VF	0.9589	0.9459	0.9524	97.40
	VT	0.900	0.9231	0.9114	97.40
	Macro avg.	0.9647	0.9673	0.9659	
	Micro avg.	0.9741	0.9741	0.9741

**Table 6:** Performance of the proposed method on fold-4, ($\mu _{NSI}$, ${V}_{NSI}$, $\mu _{NTI}$, and scale factor, $\lambda _1 = 6,\,\, \lambda _2 = 1, \,\,\lambda _3 = 1$ cases).

Fold no.	Group	Precision	Recall	F1-score	Accuracy (%)
Fold-4	PEA	1.0	1.0	1.0	100.0
	SR	1.0	1.0	1.0	100.0
	VF	1.0	0.9867	0.9933	99.62
	VT	0.9744	1.0	0.9870	99.62
	Macro avg.	0.9936	0.9967	0.9951	
	Micro avg.	0.9963	0.9963	0.9963	

From the experimental results, we observe that the classification accuracy of the PEA, VF, and VT is relatively low. Because these (PEA, VF, and VT) signals belong to the abnormal class, and the distribution of the abnormal class signals is closed distance for the combination of the selected best three features and showing high inter-dependence in the univariate histogram for the Mean of NSI feature as shown in Fig. 13.

**Figure 21:**
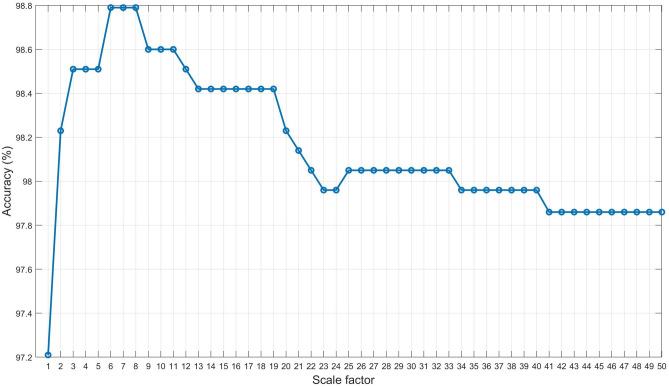
Accuracy for the different scale factor.

We have derived the detailed performance results of the proposed metric function-based decision method for the different scale factors and compared them with the Euclidean metric function and Mahalanobis metric function-based decision method. Figure 21 illustrates the summary of the performance of the proposed metric function-based decision method in terms of the different scale factors. It is observed from the figure that the highest accuracy 98.79% is obtained at $\lambda _1$ = 6, 7, 8, $\lambda _2$ = 1, $\lambda _3 = 1$, and the performance is repeated for the different scale factors. For example, the accuracy 98.51% is obtained at $\lambda _1$ = 3, 4, 5, 12, $\lambda _2$ = 1, $\lambda _3 = 1$, and the accuracy 98.79%, 98.60%, 98.42%, 98.05%, 97.96%, and 97.86% is obtained at $\lambda _1$ = 6, 7, 8, $\lambda _2$ = 1, $\lambda _3 = 1$, at $\lambda _1$ =9, 10, 11, $\lambda _2$ = 1, $\lambda _3 = 1$, at $\lambda _1$ =13 to 19, $\lambda _2$ = 1, $\lambda _3 = 1$, at $\lambda _1$ = 25 to 33, $\lambda _2$ = 1, $\lambda _3 = 1$, at $\lambda _1$ = 34 to 40, $\lambda _2$ = 1, $\lambda _3 = 1$, and at $\lambda _1$ = 41 to 50, $\lambda _2$ = 1, $\lambda _3 = 1$, respectively. The accuracy is at its peak for the different scale factors because the proposed metric function fitted well on the scatter plot by adopting different scale factors (see Fig. 13). Therefore, there is a high possibility of occurred correct classification of the test samples since open neighbors of the same groups of arrhythmias belong to the proposed metric function.

In addition, Table 7 shows the detailed performance (group-wise and different distinction schemes) comparison of the proposed metric function-based decision method and other distance-based decision methods. First, the proposed method is compared with the Euclidean metric function-based decision method regarding group-wise and different distinction schemes. As shown in the tables, 1.0 precision, 1.0 recall, 1.0 F1-score, and 100% accuracy are obtained by both methods for SR test data, while the precision, recall, F1-score, and accuracy improved for the PEA, VF, and VT test data by the proposed method. Also, the ratio of the successful discrimination between normal signals (SR) and abnormal signals (PEA, VF, and VT) is 100% for both methods, while accuracy improved for the shockable (VF, VT) versus non-shockable (PEA) arrhythmia cases by the proposed method. For example, 94.72% accuracy is achieved by the Euclidean metric function-based decision method. In contrast, the proposed metric function-based decision method increases the accuracy to 97.78%, with a 3.06% gain for the shockable (VF, VT) versus non-shockable (PEA) arrhythmia cases. In addition, the proposed method is compared with the Mahalanobis metric function-based decision method regarding different distinction schemes. The Mahalanobis metric function-based decision method achieves 86.03% accuracy for the shockable (VF, VT) and non-shockable (PEA) arrhythmias in abnormal class signals^11^. In contrast, the proposed metric function-based decision method increases the accuracy to 97.78% with an 11.75% gain.

**Table 7:** Performance comparison of the proposed metric function-based decision method with others metric function-based decision methods.

Method	Group	Precision	Recall	F1-score	Group-wise accuracy (%)	$\dag$Distinction scheme	Accuracy(%)
Proposed metric function-based decision method	PEA	0.9852	0.9925	0.9888	99.72	SR vs (PEA, VF, VT)	100.0
	SR	1.0	1.0	1.0	100.0		
	VF	0.9865	0.9799	0.9832	99.07		
	VT	0.9551	0.9613	0.9582	98.79	PEA vs (VF, VT)	97.78
	Macro avg.	0.9817	0.9834	0.9826			
	Micro avg.	0.9880	0.9880	0.9880			
Euclidean metric function-based decision method	PEA	0.9697	0.9552	0.9624	99.07	SR vs (PEA, VF, VT)	100.0
	SR	1.0	1.0	1.0	100.0		
	VF	0.9572	0.9732	0.9652	98.05		
	VT	0.9079	0.8903	0.8990	97.12	PEA vs (VF, VT)	94.72
	Macro avg.	0.9587	0.9547	0.9567			
	Micro avg.	0.9713	0.9713	0.9713			
Mahalanobis metric function-based decision method^11^						SR vs (PEA, VF, VT)	*100.0
						PEA vs (VF, VT)	86.03

$\dag$ Normal (SR) vs Abnormal (PEA, VF and VT) and Shockable (VF, VT) vs non-shockable (PEA)

*The accuracy has been calculated according to their predicted result (see part A of section III in^11^).

The performance is improved by the proposed metric function-based decision method for shockable versus non-shockable cases because we can select the best area of the scatter plot by adopting different scales of the proposed metric function. On the other hand, the performance is low of the Euclidean metric function-based and the Mahalanobis metric function-based decision method for shockable versus non-shockable cases because the Euclidean metric function and the Mahalanobis metric function are not suitable for the separation of the different groups of arrhythmias due to the characteristics of our actual scatter plot (see Fig. 13).

## Discussion

The objective of this experiment is to certify the effectiveness of our proposed method in an absolute sense and to compare relatively the performance with the existing state-of-the-art shockable and non-shockable arrhythmia discrimination methods. Table 8 shows the performance results where several factors have been considered to compare the proposed method with other methods. For example, we have compared the proposed method with other distance-based decision methods (e.g., Euclidean distance, Mahalanobis distance), and baseline methods where the same strategy was used for the information extraction from the signals. We further compared the proposed method with other existing state-of-the-art methods, which exactly followed the same databases, the same distinction scheme, and included the same arrhythmia types.

First, we compare the proposed method with the existing state-of-the-art method that exactly followed the same strategy for the information extraction from the signals. For example, Rahman et al.^17^ represented a method to derive the scalogram in the time-frequency domain. In this paper, the authors presented various experimental scalograms of the electrocardiograms using wavelet transform with various pseudo-differential-like operators and non-linear transformation functions. Then, the scalogram is analyzed only in the frequency direction, and calculated statistical features from the scalogram. Finally, the histogram is used in the decision stage to distinguish shockable and non-shockable arrhythmia. The authors achieved 100% accuracy for normal (SR) versus abnormal (PEA, VF, and VT) signals, while 91.58% accuracy was achieved for the shockable (VF, and VT) versus non-shockable (PEA) of the abnormal class signals. On the other hand, the proposed work followed the same strategy for the derivation of the scalogram from the signals and analyzed the scalogram along the frequency direction. In addition, the scalogram is analyzed along the time direction which is a new addition to our research. Also, in this proposed work we have designed a simple distance-based decision method with a scale factor where the highest accuracy is achieved. However, the proposed work achieved 100% accuracy for normal (SR) versus abnormal (PEA, VF, and VT) signals, while 97.78% accuracy was achieved for the shockable (VF, and VT) versus non-shockable (PEA) of the abnormal class signals at scale factor $\lambda _1$ = 6, $\lambda _2$ = 1, $\lambda _3$ = 1.

We further compare our proposed method with other distance-based decision methods. From Table 8 it is clear that the proposed metric function-based decision method performed better than the other distance-based decision methods. For example, in^24,25^, they used the Euclidean metric function-based decision method to distinguish arrhythmias. There it is mentioned that 91.75% and 91.67% accuracy have been obtained, while the proposed metric function-based decision method increases the accuracy to 97.78% with 6.03% and 6.11% gain. In addition, Okai et al.^11^ showed the detailed performance results of shockable versus non-shockable arrhythmia recognition algorithms by analyzing different spectrum feature parameters. They applied the Gabor wavelet transform to extract the information from the ECG signal, and used the Mahalanobis distance in their decision stage. Note that, the classification through the Mahalanobis distance depends on the concept of an approximation by means of the Gaussian distributions. The Mahalanobis metric function-based decision method achieves 100% accuracy for the distinction between normal (SR) and abnormal (PEA, VF, and VT) cases, and 86.03% accuracy for the shockable (VF, VT) and non-shockable (PEA) arrhythmias in abnormal class signals, while the proposed metric function-based decision method achieves 100% accuracy for the distinction between normal (SR) and abnormal (PEA, VF, and VT) cases and increases the accuracy to 97.78% with 11.75% gain for the shockable (VF, VT) and non-shockable (PEA) arrhythmias in abnormal class signals.

**Table 8:** Comparison of the proposed method with other state-of-the-art methods.

References (year)	Methods	Dataset used	Sample length	Group wise sample number	Distinction scheme	Performance
Tripathy et al.^12^ (2016)	VMD, RF classifier	MITDB, VFDB, CUDB	5 s	$\dag$ NSR, VF, VT, others = 1250	Shockable (VF, VT) vs non- shockable (NSR, others)	Acc = 97.23%
Cheng et al.^30^ (2017)	Personalized features, SVM	MITDB, VFDB, CUDB	8 s	VA = 1047, non-VA = 15517	VA vs Non-VA, excluded PEA	Acc = 95.46%
Acharya et al.^31^ (2018)	Pre-processing, CNN	MITDB, VFDB, CUDB	2 s	$\dag$ NSR, PEA, others = 48,095, VF, VT = 6001	Shockable (VF, VT) vs non-shockable (NSR, PEA, others)	Acc = 93.18%
Tripathy et al.^32^ (2018)	DTFT, LS-SVM	VFDB, CUDB	5 s	$\dag$ NSR, others = 4144, VF, VT = 2072	Shockable (VF, VT) vs non-shockable (NSR, others), VF vs non-VF	Acc = 83.63% Acc = 89.81%
Resiandi et al.^24^ (2018)	Preprocessing, features, KNN (K = 1 to 11)	MITDB-AFBD, NSRDB	10 s	NSR = 1280, AF = 2500	Normal vs AF	Acc = 91.75% to 78.0%
Xie et al.^33^ (2019)	SVM and Opt-AMSA	MITDB, VFDB	1 s	NSR = 50, VF = 40, VT = 58	Shockable (VF, VT) vs non-shockable (NSR)	Acc = 94.9%
Li et al.^34^ (2019)	Markov model	MITDB, VFDB, CUDB	5 s	$\dag$Shockable and non-shockable = 1670	VA vs non-VA excluded PEA	*Acc = 90.03%
Sharma et al.^10^ (2020)	Wavelet based features, (FE, RE), SVM	MITDB, VFDB, CUDB	2 s	$\dag$ NSR, PEA, VF, VT, others = 500	Shockable (VF, VT) vs non- shockable (NSR, PEA, others)	Acc = 97.8%
Okai et al.^11^ (2020)	GWT, spectrum features, Mahalanobis distance	AHA, MITDB, CUDB, KUH	5 s	SR = 552, PEA = 224, VF, VT = 356	Shockable (VF, VT) vs non-shockable (PEA), SR vs PEA, VF, VT	*Acc = 86.03% *Acc = 100.0%
Hajeb et al.^35^ (2021)	Filtering, Machine learning (BP)	MITDB, VFDB, CUDB, SDDB	14 s	$\dag$ NSR, others = 2600, VF, VT = 2340	Shockable (VT, VF) vs non-shockable (NSR, others)	Acc = 89.2%
Hammad et al.^36^ (2021)	Preprocessing, Features, PCA, SVM	MITDB, VFDB, CUDB	2 s, 5 s	$\dag$ PEA, others = 6210, VF, VT = 5794	Shockable (VF, VT) vs non-shockable (PEA, others)	Acc = 87.95% Acc = 90.14%
Toulni et al.^25^ (2021)	DWT, Features, KNN (K = 1 to 7)	MITDB	1 m	–	Normal vs abnormal	Acc = 91.67 to 66.67%
Rahman et al.^17^ (2022)	Wavelet transform pseudo differential like operators, NSI, Histogram	MITDB, VFDB, CUDB	5 s	SR = 491, PEA = 134, VF = 299, VT = 155	Shockable (VF, VT) vs non-shockable (PEA), SR vs PEA, VF, VT	Acc = 91.58% Acc = 100.0%
Proposed approach 2023	Wavelet transform pseudo differential like operators, NSI, NTI, Open neighbourhood topology	MITDB, VFDB, CUDB	5 s	SR = 491, PEA = 134, VF = 299, VT = 155	Shockable (VF, VT) vs non-shockable (PEA), SR vs PEA, VF, VT	Acc = 97.78% Acc = 100.0%

* The accuracy has been calculated according to their predicted result

$\dag$ Mentioned shockable and non-shockable sample numbers

- Not mentioned the sample number.

We also further compare our proposed method with other existing state-of-the-art methods those exactly followed the same types of distinction scheme and included PEA arrhythmia. Sharma et al.^10^ employed five-level decomposition of the signal, extracted fuzzy entropy (FE), renyi entropy (RE) features, and then fed features into various machine-learning based classifiers for the shockable and non-shockable classification. They achieved 97.8% accuracy for the Shockable (VF, VT) versus non-shockable (NSR, PEA, others), while the proposed method achieves 97.78%. The accuracy is slightly high for the existing method since the evaluation was performed on around five hundred samples where non-shockable samples (e.g., NSR samples) numbers are relatively higher than the shockable samples. In addition, we observe from Table 8 the methods as^12,30–35^, and^36^ achieved the high-performance results for shockable versus non-shockable arrhythmia distinction, but PEA arrhythmia is not individually considered there. As has been explained in the introduction the discrimination of PEA arrhythmia is particularly important in the abnormal classes regarding the actual application of AED. From the tables, we see that our proposed method obtains an accuracy comparable to or greater than the above methods with respect to the delicate distinction between shockable and non-shockable cases.

## Conclusion

Two important aspects related to the design of an arrhythmia diagnosis system of the AED have attracted the attention of this paper: how accurately does AED diagnose the shockable and non-shockable arrhythmias in the abnormal classes?; and how quickly can make a decision?; The most challenging scenario for the AED is the discrimination between non-shockable PEA and shockable VF, VT arrhythmias in the abnormal classes signals, as both signals show an unorganized electrical activity and may have similar visual characteristics. The rapid decision of AED for the application of defibrillation to arrhythmia patients increases the survival rate. From these points of view, we enhanced the arrhythmia diagnosis system in the AED. Numerical experimental results on datasets show the efficiency of the proposed methods for shockable and non-shockable arrhythmias distinction in the abnormal classes.

In this paper, we have considered four types of arrhythmias (e.g., SR, PEA, VF, and VT) to evaluate our proposed arrhythmias diagnosis system. Classifying all types of arrhythmias is essential so the clinician can prevent and treat the life-threatening ones. Therefore, all types of arrhythmias will be considered in our future work to validate our arrhythmias diagnosis system. Besides, our arrhythmias diagnosis system’s current stage remains at the software algorithms level. Therefore, the final aim of our work is to design a hardware platform that can be integrated with the AED to prevent sudden cardiac death caused by fatal arrhythmia. In this case, it is possible to translate the proposed algorithms (e.g., derivation of the scalogram, analysis of the scalogram, design of the AED shock non-shock advice algorithm) into a single hardware framework.

In addition, our future research subjects include the extraction of more effective feature parameters such as entropy-based features (e.g. Shannon entropy, Renyi entropy), Poincare plot, and so on for improving distinction accuracy. Furthermore, we will adopt SVMs (Support Vector Machines) with some kernel functions as a classifier, and evaluate their performance.

## Data Availability

The ECG dataset used in this study is available at https://archive.physionet.org/cgi-bin/atm/ATM.

## Code availability

Codes which were developed in Matlab are available from the corresponding author upon reasonable request.
